# An Exploration of Wearable Device Features Used in UK Hospital Parkinson Disease Care: Scoping Review

**DOI:** 10.2196/42950

**Published:** 2023-08-18

**Authors:** William Tam, Mohannad Alajlani, Alaa Abd-alrazaq

**Affiliations:** 1 Insitute of Digital Healthcare Warwick Manufacturing Group, University of Warwick Coventry United Kingdom; 2 Weill Cornell Medicine Doha Qatar

**Keywords:** Parkinson disease, wearable devices, machine learning, hospital, secondary care, United Kingdom, scoping review

## Abstract

**Background:**

The prevalence of Parkinson disease (PD) is becoming an increasing concern owing to the aging population in the United Kingdom. Wearable devices have the potential to improve the clinical care of patients with PD while reducing health care costs. Consequently, exploring the features of these wearable devices is important to identify the limitations and further areas of investigation of how wearable devices are currently used in clinical care in the United Kingdom.

**Objective:**

In this scoping review, we aimed to explore the features of wearable devices used for PD in hospitals in the United Kingdom.

**Methods:**

A scoping review of the current research was undertaken and reported according to the PRISMA-ScR (Preferred Reporting Items for Systematic Reviews and Meta-Analyses extension for Scoping Reviews) guidelines. The literature search was undertaken on June 6, 2022, and publications were obtained from MEDLINE or PubMed, Embase, and the Cochrane Library. Eligible publications were initially screened by their titles and abstracts. Publications that passed the initial screening underwent a full review. The study characteristics were extracted from the final publications, and the evidence was synthesized using a narrative approach. Any queries were reviewed by the first and second authors.

**Results:**

Of the 4543 publications identified, 39 (0.86%) publications underwent a full review, and 20 (0.44%) publications were included in the scoping review. Most studies (11/20, 55%) were conducted at the Newcastle upon Tyne Hospitals NHS Foundation Trust, with sample sizes ranging from 10 to 418. Most study participants were male individuals with a mean age ranging from 57.7 to 78.0 years. The AX3 was the most popular device brand used, and it was commercially manufactured by Axivity. Common wearable device types included body-worn sensors, inertial measurement units, and smartwatches that used accelerometers and gyroscopes to measure the clinical features of PD. Most wearable device primary measures involved the measured gait, bradykinesia, and dyskinesia. The most common wearable device placements were the lumbar region, head, and wrist. Furthermore, 65% (13/20) of the studies used artificial intelligence or machine learning to support PD data analysis.

**Conclusions:**

This study demonstrated that wearable devices could help provide a more detailed analysis of PD symptoms during the assessment phase and personalize treatment. Using machine learning, wearable devices could differentiate PD from other neurodegenerative diseases. The identified evidence gaps include the lack of analysis of wearable device cybersecurity and data management. The lack of cost-effectiveness analysis and large-scale participation in studies resulted in uncertainty regarding the feasibility of the widespread use of wearable devices. The uncertainty around the identified research gaps was further exacerbated by the lack of medical regulation of wearable devices for PD, particularly in the United Kingdom where regulations were changing due to the political landscape.

## Introduction

### Background

In the future, the proportion of the population aged >65 years in the United Kingdom is projected to continue to increase [1,2], and this population is estimated to increase to approximately 18 million by 2037 [3]. Half of the people in this population are projected to have one or more chronic diseases [1,4]. The older population in the United Kingdom is likely to develop chronic neurodegenerative diseases, with Parkinson disease (PD) being the second most common neurodegenerative disease [5]. On the basis of the United Kingdom’s estimated PD prevalence reported in 2017, this number was estimated to rise by 18% (from 283,585 to 337,165) from 2018 to 2025 [6]. The disease burden of PD can have various clinical and socioeconomic consequences. PD care costs can increase with disease severity and progression [7,8]. Costs are commonly attributed to medication, support services, and hospital admissions [7]. Furthermore, complex care needs can negatively impact patient and carer well-being [9,10] due to reduced autonomy and increased financial strain [11]. The reduced quality of life can subsequently result in increased nursing home admissions [12]. Therefore, further research is required to develop solutions to address the emerging concerns and challenges associated with PD.

UK secondary care is mainly defined as the care taken directly in hospitals or on hospital grounds but can also include care undertaken in the community care by specialist nurses [13]. In contrast to UK primary care, which is commonly the first point of contact for patients, UK secondary care requires patient referral where the patient can then be reviewed and managed by clinical teams with more specialist knowledge [13]. Furthermore, UK secondary care is also distinct from tertiary care that provides further specialist knowledge, staff, and facilities [13]. Tertiary care provides even higher levels of specialized services to treat complex and rare diseases that are often associated with fields such as neurosurgery, cardiac surgery, and neonatology. Simple assessments of PD in primary care often take place in general practice by evaluating changes in motor function, mood, and quality of life using the Hoehn and Yar scale or the Unified Parkinson’s Disease Rating Scale (UPDRS) [14]. However, the Hoehn and Yar or UPDRS scores are subjective and can vary depending on the assessor [15]. More complex and objective assessments can be performed in hospitals. This involves attending specific assessment laboratories using specialist equipment such as pressure-sensor walkways when conducting the timed up-and-go (TUG) assessments [16].

Advances in medical technology have improved clinical assessments, patient outcomes, operational efficiency, and accessibility to clinical services [17-20]. Positive attitudes toward technology adoption in health care have been further accelerated by the COVID-19 pandemic [21-23]. The rise of wearable devices has been successful in the continuous collection and monitoring of patients’ vital signs [24]. Wearable devices are electronic devices powered by microprocessors that can be worn as accessories, embedded in clothing, or implanted in a user’s body with the ability to send and receive data [25]. Health care wearable devices are often used to monitor medical symptoms, empower patients to manage their care (eg, via medication reminders), and allow clinical teams to analyze wearable device data to improve the optimization and management of treatment [24]. Consequently, evolving technological developments and the need to adopt technology in health care during the COVID-19 pandemic have led to increasing interest in how wearable devices can be used in health care. Wearable devices have shown the potential to improve clinical outcomes, as the personalized information provided by wearable devices has resulted in more timely action, informed decision-making, and multidisciplinary collaboration [26-28].

The widespread integration of wearable devices may also further benefit clinicians, patients, and carers. Significant costs related to PD care are often attributed to medication, hospital admissions, and support services [7]. PD can also decrease the quality of life of the patient, family, and carers due to the loss of independence and negative impact on well-being [9]. However, by improving care in areas such as early diagnosis, more comprehensive and timely diagnosis can enable patients to access appropriate services and increase the efficacy of care [29]. Wearable devices have shown promise for improving and innovating PD care. As technology improves, there has been further research into how technology could be leveraged to increase the efficiency of care delivery. Improving the efficiency of care mainly involves using wearable device to enable remote monitoring and create web-based clinics to increase access to care in remote geographic locations, increase the timeliness of obtaining appointments, and simplify appointment schedules, which may reduce the strain on patients, carers, and families [15,30]. Currently, wearable devices used in PD care involve the use of accelerometers and gyroscopes that are commonly used to measure PD motor symptoms such as bradykinesia and tremor [31]. Furthermore, research has shown that there is increasing interest in the use of wearable devices to improve PD care and management in patients living with PD [32]. The rising interest in PD wearable devices has led to the development of commercial devices such as Parkinson’s KinetiGraph (PKG) [33]. The main appeal of the wrist-worn device is its ability to continuously collect data on tremors over time [33]. In PD hospital care, Rovini et al [34] identified that wearable devices were commonly used for early diagnosis, as well as body motion or freezing of gait analysis during gait and TUG assessments. Wearable devices have been shown to have success by using biomarkers such as gait to distinguish between individuals diagnosed with early signs of PD and healthy control group [35-37]. In hospital laboratory settings, wearable devices have also been used to monitor the severity of PD symptoms, and each PD symptom fluctuates throughout the day [38]. Wearable devices such as PKGs have also shown promise in improving medication compliance and management of patients with PD by using medication reminders [39]. For nonmotor PD symptoms, wearable devices may have the potential to measure moods to assist with psychotherapy; however, there has been relatively little research into how this can be achieved. Currently, wearable devices rely on monitoring the characteristics of motor PD symptoms such as gait and number of turns, with little understanding of how the data can be used to analyze the patients’ nonmotor PD symptoms [37,40,41]. The prospects of using artificial intelligence (AI) and machine learning (ML) technology with wearable devices have also been explored in the United Kingdom. Del Din et al [42] were able to successfully use algorithms to analyze gait characteristics including gait asymmetry, gait variability, stride length, and step time. Consequently, wearable devices when combined with algorithms may provide more detailed and objective data for PD assessments, as well as provide more flexibility regarding the placements of PD wearable devices rather than limit them to the L5 vertebra, which is considered the gold standard location [42]. According to previous research, wearable devices have the potential to enable clinicians to obtain more sensitive and objective data that can improve PD assessment, as well as offer an alternative to using more expensive specialist equipment and laboratories [43].

However, the main challenges with wearable device use in health care are due to the unique requirements and regulations outlined by individual countries, and thus, the findings would be less generalizable and unable to be suitably translated to other countries. In the United Kingdom, institutions such as the National Institute of Clinical Excellence (NICE), the Care Quality Commission, and the Medicine and Healthcare products Regulatory Agency often guide the development of regulations and implementation of health care technology by evaluating factors such as clinical effectiveness, cost, and safety [44-46]. By contrast, health care systems in other countries may adhere to different technical regulations; for example, Europe adheres to technical regulations such as the European General Data Protection Regulation, and the United States follows guidance from the Food and Drug Administration (FDA) and the National Institute of Standard Technology [47-49].

Due to the increasing prevalence of PD in the United Kingdom, there has also been increasing research into how wearable devices can be used to improve PD care in hospital settings. In this scoping review, we attempted to consolidate current research and explore how wearable devices are used in PD care in UK hospitals.

### Research Gap and Aim

Despite increasing positive sentiments toward technology in health care [21,22], improving clinical outcomes or practice in various clinical settings by using wearable devices remains inconclusive [50]. PD trials are often constrained by low recruitment and short-term trials [51]. The long-term performance of wearable devices also remains uncertain because their performance can vary based on patient demographics [50]. Romano and Stafford [52] highlighted the challenges of implementing key performance indicators to measure and demonstrate the proposed benefits of technology in improving clinical care and decision-making. Furthermore, analytic data can remain unreliable, as the observed accuracy of data and errors range between 9.3% and 23.5% [53].

Currently, there is no clear documentation of the types of wearable devices used in PD care and the clinical settings in which wearable devices are used. Furthermore, IT security studies have highlighted users’ lack of awareness and self-responsibility regarding wearable device information security [54,55]. However, current clinical studies have not fully addressed wearable device security or data integrity concerns. By aggregating current research via a scoping review, researchers can review current applications and limitations to understand the current wearable devices used in PD care. Due to the scope of this scoping review, we aimed to focus on exploring the features of wearable devices used in PD treatment and care within UK secondary care settings.

## Methods

### Protocol and Registration

The population, patient, or participants, interventions, comparators, and outcomes (PICO) framework [56] and the PRISMA-ScR (Preferred Reporting Items for Systematic Reviews and Meta-Analyses extension for Scoping Reviews) guidelines were followed to support the design and reporting of the scoping review (Multimedia Appendix 1). The protocol of the scoping review is shown in Multimedia Appendix 2.

### Study Eligibility Criteria

The eligibility criteria were full conference papers, peer-reviewed articles, study protocols, theses, and dissertations that focused on using wearable devices for patients with PD in UK hospital settings. Wearable devices were categorized as electronic devices, sensors, or technologies that can be worn as accessories, embedded in clothing, or implanted in users’ body, with the ability to send and receive data. Smart tattoos and traditional hearing aids were excluded because of their inability to receive and transmit information [57]. UK hospital care was defined as the care undertaken directly in hospitals and overseen by hospital clinical teams [13]. This scoping review focused on wearable device research in UK hospital settings because current gold standard PD assessments often take place in designated hospital research laboratories using specialist equipment, with wearable devices as an alternative to expensive specialist PD assessment laboratories [42,43]. Hospital settings included inpatient wards, emergency departments, outpatient clinics, and test laboratory environments situated on hospital grounds. This review excluded secondary services and primary care that were undertaken in the community setting, such as community specialist nursing [13]. Primary care was excluded because clinicians relied only on simple PD assessments such as the UPDRS and lacked adequate facilities wherein patients were commonly referred to specialist hospital PD assessment laboratories to conduct more comprehensive PD assessments [14-16]. Research conducted in tertiary care [13] relating to specialized procedures that required referrals to designated National Health Service (NHS) centers of excellence was also excluded. This scoping review included studies published in English within the past 5 years (2017 to 2022) and excluded reviews, conference abstracts, reports, and editorials. These sources were often inadequately recorded and contained unsuitable information to enable sufficient data extraction of the study characteristics of interest, or the studies were not published in full. Previous research supported that conference abstracts contained insufficient information in areas such as the study details needed for comprehensive data extraction [58]. It was decided to include only publications published within the past 5 years as technological innovations continue to improve. Technological fields such as the semiconductor industry have continued to show how quickly technological innovation can occur [59]. Material science innovations have also led to wearable devices becoming more accessible and usable by reducing costs, improving sensor detection, and miniaturizing wearable devices, thus making them less obtrusive [60]. The COVID-19 pandemic has also highlighted to medical staff and the general public how technology such as wearable devices can improve health care. This has led to further investigation into wearable device use within health care settings during the pandemic [61,62], as well as how wearable devices can continue to be used in areas such as PD treatment developments [63]. No restrictions were applied to the age, gender, and ethnicity of the participants; study design; and measured outcomes. Table 1 presents the inclusion and exclusion criteria for the study.

**Table 1:** Population, interventions, comparators, and outcomes criteria [56].

	Inclusion criteria	Exclusion criteria
Population (Parkinson disease)	Patients with a confirmed Parkinson disease diagnosis	Patients with other medical conditions
Intervention (wearable devices)	Use of wearable devices in Parkinson disease care. Wearable device applications include the tracking and monitoring of information to facilitate improving Parkinson disease clinical practice, assessment, and diagnosis Wearable devices were categorized as electronic devices, sensors, or technology that can be worn as accessories, embedded in clothing, or implanted in users’ body with the ability to send and receive data	Nonwearable devices such as portable devices including mobile phones and tablets
Outcome	No restrictions applied	No restrictions on the outcome applied
Comparator	No comparator was required. However, when comparators such as controls were identified, this was labeled appropriately. Controls included healthy age-matched controls and baseline readings	No restrictions on comparators applied
Setting (hospitals in the United Kingdom)	Parkinson disease research or care within hospitals or in specialized laboratories located at or within hospital grounds throughout the United Kingdom	Parkinson care or research taken in community settings such as general practice, community care homes, or patients’ home Care or research taking place outside of the United Kingdom
Publication type	Full conference papers, peer-reviewed articles, study protocols, theses, and dissertations	Reviews, conference abstracts, reports, and editorials
Publication language	English only	Non-English texts
Publication year	2017-2022	Texts published outside of 2017-2022

### Information Sources

On June 6, 2022, the first author searched 3 main databases: PubMed or MEDLINE, Embase (via Ovid), and the Cochrane Library. The PubMed or MEDLINE database captured peer-reviewed academic journals related to the health care field, whereas Embase contained publications related to medical devices, as well as biomedical and pharmacological research. The Cochrane Library was also searched to capture medical clinical trials and Cochrane reviews. Inclusion of the selected databases increased the probability that the literature was diverse, representative, and contained a wide variety of study designs and research topics. Due to time and staffing limitations, as well as the number of search results, forward and backward reference list checking was not conducted. Databases were restricted to established peer-reviewed databases to ensure a minimum standard of quality and credibility of the publications included [64,65]. We decided to follow the recommendations of Cochrane to focus on established peer-reviewed databases [66].

### Search

The search query was developed by the first author and reviewed by the second author, who has extensive experience in systematic reviews. Search terms were developed from the identified key research concepts: wearable devices, PD, and UK secondary care settings. The title and abstract search terms were used to identify relevant publications. Medical Subject Headings for PD or PD symptoms (eg, gait or bradykinesia) were used to capture relevant publications. Truncation was considered to identify alternative word variations of the key research concepts such as *Parkinson’s disease* and *parkinsonism*, which may be included in different publications. The search strategy used for the scoping review is available in Multimedia Appendix 3.

### Selection of Sources of Evidence

The eligible studies were identified and selected by the first author. All retrieved studies were exported to Zotero (Corporation for Digital Scholarship) where duplicates were identified using the duplicate item function and removed. The publications were then screened by the title and abstract by the first author. Publications that passed the initial screening were then read in full by the first author. Any doubts regarding the eligibility of the identified publications were resolved following a discussion with the second author.

### Data Extraction

The data extraction form was developed based on recommendations provided by the second author. Data were extracted from the included publications by the first author. The extracted data included information about the study metadata (eg, year of publication, study location, hospital, publication type, sample size, gender ratio of participants, age, PD duration, and PD severity, as well as if any control groups were used); features of wearable devices used (eg, device brand, manufacturer, commercial or noncommercial device, approval of the wearable devices for clinical use by regulators, wearable device aim, wearable device sensor, type of device, wearable device location, the biosignal measured, connectivity, the host device, sensing approach, and length of time the wearable device was used in the study); the type of AI and ML technology used (eg, least absolute shrinkage and selection operator, support vector machine, and random forest); and the clinical aim of AI and ML technology used. The extracted data were then checked by the second author, and any discrepancies were resolved by discussion. A list of the extracted features is available in Multimedia Appendix 4.

### Synthesis of Results

Narrative methods were used to summarize and synthesize the collected data, which followed the constructivist approach to analyze the studies and identify research gaps [67,68]. Constructivism is a framework to critically analyze research findings, highlight current knowledge gaps, and establish study limitations [67]. Furthermore, by using a constructivist approach in narrative synthesis, existing research can be developed by discussing the implications of the current findings and concerns [68]. A combination of text, tables, and graphs was used to describe the study characteristics of the final included publications. Graphs were used to present key data such as the date of publication, type of study design and outcome of each publication; the application of the wearable device used in the study; and the type of wearable device used.

## Results

### Search Results

**Figure 1:**
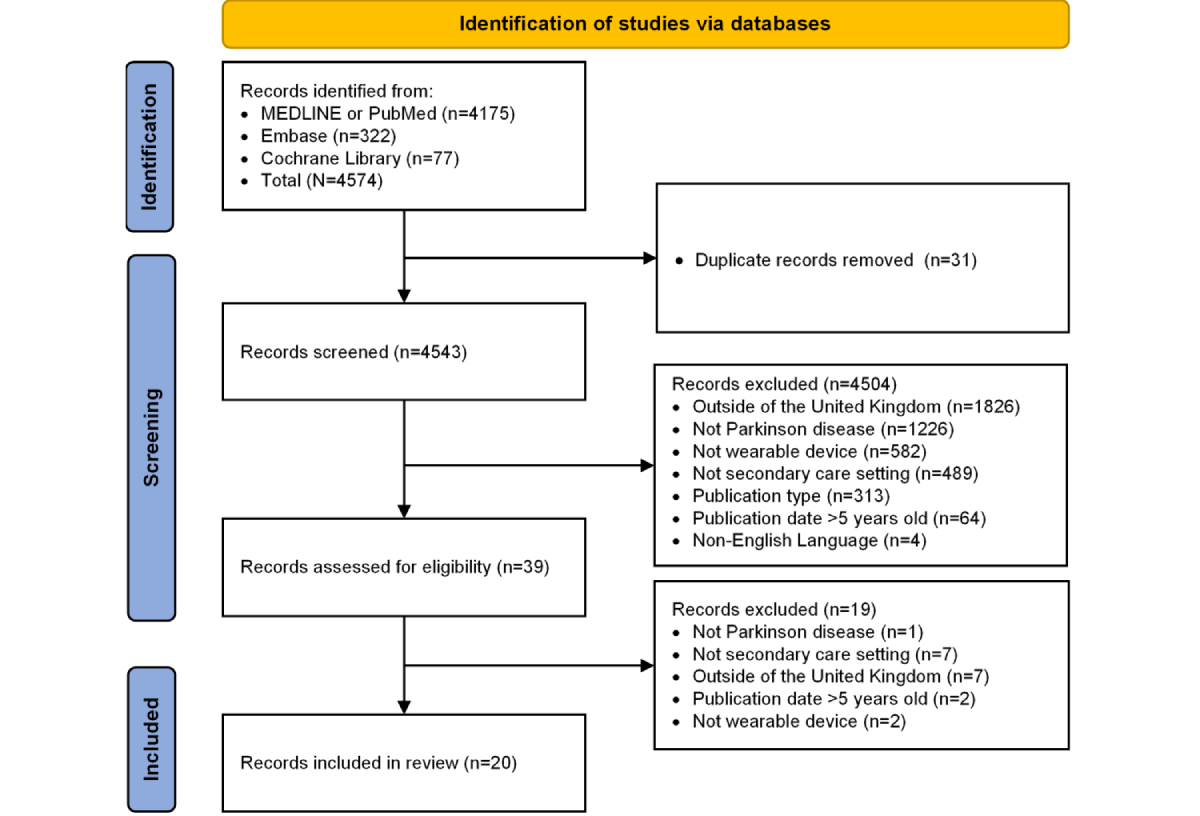
PRISMA (Preferred Reporting Items for Systematic Reviews and Meta-Analyses) flowchart outlining the initial screening process.

A total of 4574 publications were identified, and with 31 (0.68%) duplicates removed, 4543 (99.32%) unique publications were obtained (Figure 1). Of these, 4504 (99.14%) publications were excluded during the initial screening of the titles and abstracts for the reasons reported in Figure 1. Of the remaining 39 publications, 19 (49%) were excluded after the full-text review, and a total of 20 (51%) eligible publications were included in this review.

### General Description of Included Studies

Table 2 presents the general characteristics of the included publications. The included publications were published between 2016 and 2021. Most of the studies were published in 2019 (6/20, 30%) and 2020 (6/20, 30%). All included publications (20/20, 100%) were journal articles. Studies were conducted in Newcastle (11/20, 55%), London (3/20, 15%), Leeds (2/20, 10%), and Oxford (2/20, 10%); the remaining studies were conducted in Plymouth, Sheffield, and Southampton. All the studies were conducted in secondary hospital settings or research facilities on hospital grounds, with most studies (11/20, 55%) conducted at the Newcastle upon Tyne Hospitals NHS Foundation Trust. Huo et al [69] and Payne et al [70] conducted trials across multiple sites within the United Kingdom, accounting for more than 20 locations. The number of participants was >100 in 55% (11/20) of the studies, between 51 and 100 in 15% (3/20) of the studies, and <50 in 30% (6/20) of the studies. The average sample size was 108 (SD ±91.1). Among the studies that documented the ratio of male to female participants (16/20, 80%), the percentage of males ranged from 21% to 100%, with an average of 58.1%. In the studies that documented the mean age (16/20, 80%), most participants were older individuals, with 75% (12/16) of studies recording a mean age of >65 years. The PD duration of patients with PD after the initial confirmed diagnosis ranged widely from approximately 4 months to 24 years. On the basis of the UPDRS categories, 65% (13/20) of the studies reported patients with moderate to severe PD [71], whereas 40% (8/20) of the studies included patients with moderate PD, and 35% (7/20) of the studies included patients who were reported to have severe PD. No correlation or observation could be made if wearable device use increased with increasing PD severity. In both the studies conducted by Mc Ardle et al [72,73], study participants were ineligible or discontinued the trial partway through; therefore, a lower total number of male and female study participants was recorded. The mean age varied between 57.7 and 78.0 years, with a total combined average age of 68.2 years.

**Table 2:** General study characteristics of included publications.

Study, year	Study location	Hospital	Publication type	Sample size, n	Male:female ratio	Age (years), mean (SD)	Parkinson disease duration (years), mean (SD)	Parkinson disease severity	Control
Buckley et al [74], 2019	Newcastle	Newcastle upon Tyne Hospitals NHS^a^ Foundation Trust	Journal article	134	82:52	70.4 (SD 8.4)	≤0.33 (SD not reported)	Not reported	Healthy age-matched controls
Coates et al [75], 2020	Newcastle	Newcastle upon Tyne Hospitals NHS Foundation Trust	Journal article	10	10:0	67.8 (SD 9.0)	Not reported	Mild to moderate	Healthy age-matched controls
De Vos et al [76], 2020	Oxford	John Radcliffe Hospital	Journal article	80	42:38	66.4 (SD not reported)	11.4 (SD not reported)	Moderate	Healthy age-matched controls
Dominey et al [77], 2020	Plymouth	Plymouth Hospital	Journal article	166	95:71	70 (SD not reported)	6 (SD not reported)	Not reported	Not reported
Dunne-Willows et al [78], 2019	Newcastle	Newcastle upon Tyne Hospitals NHS Foundation Trust	Journal article	79	31:48	67.4 (SD 8.5)	Not reported	Mild	Healthy age-matched controls
Huo et al [69], 2020	Oxford	Charing Cross Hospital John Radcliffe Hospital	Journal article	33	20:13	57.7 (SD 10.4)	11.1 (SD 5.5)	Mild to severe	Healthy nonage-matched controls
Lacy et al [79], 2018	Leeds	Leeds General Infirmary	Journal article	177	Not reported	Not reported	Not reported	Not reported	Healthy age-matched controls
Lones et al [80], 2017	Leeds	Leeds General Infirmary	Journal article	23	15:8	68.0 (SD 8.1)	9.0 (SD 3.7)	Moderate	Not reported
Mc Ardle et al [72], 2019	Newcastle	Newcastle upon Tyne Hospitals NHS Foundation Trust	Journal article	80	50:24	78.0 (SD 6.0)	Not reported	Severe	Healthy age-matched controls
Mc Ardle et al [73], 2021	Newcastle	Newcastle upon Tyne Hospitals NHS Foundation Trust	Journal article	125	59:38	76.5 (SD 6.8)	Not reported	Severe	Healthy age-matched controls
Pantall et al [81], 2018	Newcastle	Newcastle upon Tyne Hospitals NHS Foundation Trust	Journal article	109	66:43	70.9 (SD 7.5)	Not reported	Moderate	Healthy age-matched controls
Pantall et al [82], 2018	Newcastle	Newcastle upon Tyne Hospitals NHS Foundation Trust	Journal article	35	23:12	65.9 (SD 8.2)	0.45 (SD 0.3)	Moderate to severe	Baseline reading
Payne et al [70], 2020	Sheffield London	Sheffield Teaching Hospitals NHS Trust University College London Hospitals NHS Foundation Trust	Journal article	30	Not reported	Not reported	≤3.0 (SD not reported)	Mild	Placebo control
Rehman et al [83], 2019	Newcastle	Newcastle upon Tyne Hospitals NHS Foundation Trust	Journal article	196	108:88	62.9 (SD 8.4)	23.8 (SD 4.2)	Severe	Healthy age-matched controls
Rehman et al [84], 2019	Newcastle	Newcastle upon Tyne Hospitals NHS Foundation Trust	Journal article	142	87:55	69.7 (SD 8.9)	24.1 (SD 4.8)	Severe	Healthy age-matched controls
Rehman et al [85], 2020	Newcastle	Newcastle upon Tyne Hospitals NHS Foundation Trust	Journal article	93	58:35	70.1 (SD 8.2)	3.1 (SD 0.2)	Severe	Healthy age-matched controls
Stack et al [86], 2018	Southampton	Southampton General Hospital	Journal article	24	5:19	74.0 (SD not reported)	Not reported	Not reported	Healthy age-matched controls
Stuart et al [87], 2016	Newcastle	Newcastle upon Tyne Hospitals NHS Foundation Trust	Journal article	100	Not reported	Not reported	Not reported	Not reported	Healthy age-matched controls
van Wamelen et al [88], 2019	London	King’s College London Hospital	Journal article	108	72:36	62.8 (SD 9.4)	7.5 (SD 5.5)	Moderate	Baseline reading
van Wamelen et al [89], 2021	London	King’s College London Hospital	Journal article	418	273:145	62.7 (SD 8.5)	4.2 (SD 11.67)	Mild to moderate	Baseline reading

^a^NHS: National Health Service.

Healthy age-matched controls were used by 70% (14/20) of the studies. Wearable devices identified differences in motor symptom characteristics in patients with PD compared with healthy age-matched controls and patients with other neurodegenerative diseases such as progressive supranuclear palsy (PSP). This allowed clinicians to provide more detailed PD assessments for their patients compared with controls and potentially develop more personalized care or improve the timeliness of early diagnosis in the future. Wearable devices were able to identify significant differences in gait between patients with PD and healthy controls by characteristics such as swing time, step length, and step asymmetry [76,78,85]. In addition, wearable devices could also be used to identify other biomarkers that could aid in PD assessment. Coates et al [75] identified that patients with PD showed statistically significantly higher levels of sample entropy and found that sample entropy was significantly correlated with levodopa equivalent daily dose but not with UPDRS scores. Buckley et al [74] determined that only particular spatiotemporal features were able to significantly discriminate between patients with PD and healthy controls; for example, step irregularity was the best discriminator when using pelvis acceleration, and pace characteristics were able to further help classify gait in patients with PD compared with healthy controls using upper body and pelvis acceleration. Using wearable devices to investigate nonmotor symptoms was more challenging. Pantall et al [81] were only able to identify a weak negative correlation between sample entropy and cognitive characteristics; however, the wearable device could not be used to provide more information and would require further research using neuroimaging to confirm any correlation. The main benefit of using wearable devices was the ability to receive medication reminders to improve medication management, and the patients were happy to continue using the device due to its convenience [77]. The consensus remained divided on whether wearable devices could provide any benefit by decreasing the number of appointment attendances. Some patients were satisfied that they did not have to travel to clinics as often, but others were neutral about this issue [77]. Current data only used patient satisfaction surveys, and no statistical analyses were conducted to assess whether wearable devices reduced the need for clinical visits.

### Features of Wearable Devices

Table 3 presents the general features of the wearable devices used in each study. Most studies (18/20, 90%) used a single device brand, whereas the remaining studies (2/20, 10%) used ≥2 device brands. The AX3 accelerometer was the most popular device brand used (9/20, 45%). Consequently, Axivity was the most common wearable device manufacturer. The AX3 and PKG wearable devices also contained light and temperature sensors. The light sensor can be used to measure different frequencies of light, and the temperature sensor can measure the temperature of the environment [90]. Most studies (17/20, 85%) used commercially available devices manufactured by various technology companies, and only 15% (3/20) of the studies created prototype devices. Wearable devices were used to monitor PD symptoms (18/20, 90%) or potential biomarkers (2/20, 10%). Accelerometers (19/20, 95%) were the most commonly used sensors in wearable devices, followed by gyroscopes (7/20, 35%) to monitor PD symptoms and biomarkers.

In 55% (11/20) of the studies, the manufacturer did not report whether the devices were approved by any regulatory body. Most notably, this was related to the use of the AX3 that is manufactured by Axivity. In the other 9 (N=20, 45%) studies in which the regulatory approval was reported, the wearable device was approved by the FDA, Conformité Européenne (CE) medical mark approval, or both (Table 3).

Table 4 highlights the technical features of wearable devices and shows that most studies used smart bands (13/20, 65%), followed by inertial measurement units (IMUs; 4/20, 20%) and smartwatches (3/20, 15%). All the publications that used smartwatches used the PKG, which contains an accelerometer built into the smartwatch [88]. Only Huo et al [69] developed a bespoke wearable device that included monitoring sensors; inertial measurement devices (containing an accelerometer, gyroscope, and magnetometer); and force sensors to capture muscle stiffness and activity in patients with PD. In addition, Huo et al [69] used mechanomyography sensors in the bespoke device, which are distinct from the electromagnetic sensors used by Lacy et al [79]. Electromagnetic sensors were used to detect electrical activity in response to nerve stimulation of a muscle fiber, whereas mechanomyography sensors were used to detect muscular vibrations that occurred during muscle contraction [91].

The most common wearable device placement was on the lumbar region (11/20, 55%), followed by the head (5/20, 25%), and wrist (5/20, 25%). Some studies (9/20, 45%) used wearable devices to monitor and measure 1 primary biosignal, and 55% (11/20) of the studies used wearable devices to monitor multiple characteristics. All the studies (20/20, 100%) investigated PD motor symptoms. Table 4 shows that the primary biosignal measured by wearable devices was gait (10/20, 50%), followed by bradykinesia (4/20, 20%) and dyskinesia (3/20, 15%). Primary nonmotor symptom measures (1/20, 5%) were relatively unexplored and were monitored alongside motor symptoms (Table 4).

**Table 3:** General features of wearable devices.

Study, year	Device brand	Manufacturer	Wearable device status	Regulatory approval for clinical use	Aim of the wearable device use	Length of time wearable device was used
Buckley et al [74], 2019	Opal	APDM^a^	Commercial	FDA^b^ CE^c^ medical mark	Monitoring PD^d^ biomarkers	Single session
Coates et al [75], 2020	AX3	Axivity	Commercial	Not reported	Monitoring PD biomarkers	Single session
De Vos et al [76], 2020	Opal	APDM	Commercial	FDA	Monitoring PD symptoms	Single session
Dominey et al [77], 2020	Parkinson’s KinetiGraph	Global Kinetics	Commercial	FDA CE medical mark	Monitoring PD symptoms Medication reminder alerts	Multiple sessions over time
Dunne-Willows et al [78], 2019	AX3	Axivity	Commercial	Not reported	Monitoring PD symptoms	Single session
Huo et al [69], 2020	Bespoke	Bespoke	Noncommercial	Not reported	Monitoring PD symptoms	Single session
Lacy et al [79], 2018	PD-Monitor	ClearSky	Noncommercial	CE medical mark	Monitoring PD symptoms	Single session
Lones et al [80], 2017	LID^e^-Monitor	ClearSky	Commercial	CE medical mark	Monitoring PD symptoms	Single session
Mc Ardle et al [72], 2020	AX3	Axivity	Commercial	Not reported	Monitoring PD symptoms	Single session
Mc Ardle et al [73], 2021	AX3	Axivity	Commercial	Not reported	Monitoring PD symptoms	Single session
Pantall et al [81], 2018	AX3	Axivity	Commercial	Not reported	Monitoring PD biomarkers	Multiple sessions over time
Pantall et al [82], 2018	AX3	Axivity	Commercial	Not reported	Monitoring PD symptoms	Multiple sessions over time
Payne et al [70], 2020	Opal Dynaport Movemonitor	APDM McRoberts	Commercial	FDA CE medical mark	Monitoring PD symptoms	Multiple sessions over time
Rehman et al [83], 2019	AX3	Axivity	Commercial	Not reported	Monitoring PD symptoms	Single session
Rehman et al [84], 2020	AX3	Axivity	Commercial	Not reported	Monitoring PD symptoms	Single session
Rehman et al [85], 2020	Opal	APDM	Commercial	CE medical mark	Monitoring PD symptoms	Single session
Stack et al [86], 2018	Not reported	Not reported	Noncommercial	Not reported	Monitoring PD symptoms	Single session
Stuart et al [87], 2015	AX3 Dikablis	Axivity Ergoneers	Commercial	Not reported	Monitoring PD symptoms	Multiple sessions over time
van Wamelen et al [88], 2019	Parkinson’s KinetiGraph	Global Kinetics	Commercial	FDA CE medical mark	Monitoring PD symptoms	Multiple sessions over time
van Wamelen et al [89], 2021	Parkinson’s KinetiGraph	Global Kinetics	Commercial	FDA CE medical mark	Monitoring PD symptoms	Multiple sessions over time

^a^APDM: Ambulatory Parkinson’s Disease Monitoring.

^b^FDA: Food and Drug Administration.

^c^CE: Conformité Européenne.

^d^PD: Parkinson disease.

^e^LID: levodopa-induced dyskinesia.

The studies attempted to identify quantifiable biomarkers that could be measured with wearable devices to provide more information about a patient’s PD state, as well as to determine how biomarkers and symptoms contributed to the overall patient condition (Table 4). Many biomarkers and symptoms were related to the measurement of motor symptoms and physical and clinical features. Common biomarkers and symptoms included bradykinesia; dyskinesia; gait characteristics (eg, step asymmetry, stride length, step velocity, and swing time); tremor; and hand rigidity. Wearable devices with built-in accelerometers measured fluctuations in movement and acceleration over time [72,78,83-85]. Wearable devices such as the PKG also contained built-in accelerometers to measure biomarkers such as bradykinesia and dyskinesia, and the algorithms have been validated against established frameworks such as the UPDRS [69,92]. Multiple studies found that wearable devices could be used to measure and analyze gait characteristics to successfully discriminate patients with PD from healthy controls and other neurodegenerative diseases [72,78,83-85]. Although current studies have shown that gait characteristic data could be used as potential biomarkers to support PD assessment, biomarkers such as gait characteristic analysis have yet to be comprehensively validated due to the lack of formal criteria, and the studies instead relied on previous research results or potentially subjective criteria [78,83,93]. In contrast, thresholds determining when patients require clinical intervention relied on clinicians interpreting PKG data and determined when medication doses were becoming ineffective and when the dose required adjustments [77]. There was less focus on using wearable devices to measure and interpret nonmotor symptoms such as mood and cognition. Wearable devices could only be used to identify if nonmotor symptoms were correlated with other clinical features but could not further the clinical context or the significance of these readings [77]. Furthermore, the current identified studies did not determine how cognitive function influenced motor symptoms such as gait [86].

Table 4 shows that most studies used USB cables (9/20, 45%) and Wi-Fi connections (9/20, 45%) to connect the wearable device to the host device. Stuart et al [87] used an AX3 and a Dikablis eye tracker, which can be connected via a USB cable and Wi-Fi connection, accounting for more than 20 data points. The overwhelming majority of the collected wearable device information was stored on data servers (18/20, 90%). All the studies (20/20, 100%) used an opportunistic sensing approach as presented in Table 3. The wearable device was used for a single session at the hospital in 65% (13/20) of the studies. In 35% (7/20) of the studies, the study duration ranged from 6 days to 54 months and included multiple separate sessions using the wearable device at the hospital assessment center. This was insufficient to determine if the wearable devices were medically prescribed to the study participants, which is compounded by the uncertainty around the official regulation, distribution, and use of wearable devices for patients with PD in the United Kingdom [94].

In this review, AI and ML algorithms were categorized into classification models, regression models, neural network–based models, and optimization algorithms (Table 4). Algorithms that were documented as being used in the study but not specified by the study authors were labeled as a black box. Categorization and definitions were created according to the suggestion of the corresponding author, AA-a. The types of AI and ML algorithms used in the included studies are presented in Table 4. Of the studies that used AI and ML (17/20, 85%), most (13/17, 76%) used 1 algorithm or model. Lacy et al [79] and Rehman et al [83] compared 2 algorithms or models during tests, whereas Huo et al [69] used multiple classification models to develop a bespoke classification system and improve upon existing models. The black box and area under the curve (AUC) algorithms (4/17, 24%) were the most reported AI and ML technologies used, followed by the random forest model (2/17, 12%) and the partial least square discriminant analysis model (2/17, 12%). The applications of AI and ML mainly revolved around PD classification (9/17, 53%). PD classification primarily involved algorithms that were able to distinguish patients with PD from other patients with neurodegenerative diseases such as Alzheimer disease (AD) or healthy controls, in addition to using algorithms to identify and analyze clinical features to improve PD classification. In 35% (6/17) of studies, algorithms were used to determine if the measured biosignal correlated with PD clinical features such as PD motor symptoms or medication dose, and 29% (5/17) of the studies specifically analyzed gait and used algorithms to provide greater detail and information about gait characteristics such as step velocity and turn characteristics. Only Huo et al [69] used algorithms to rank PD symptoms and determine how each symptom contributed to the overall PD severity score.

**Table 4:** Technical characteristics of wearable devices.

Study, year	Sensor	Wearable device type	Placement of wearable device	Measured biosignal	Connectivity	Host device	Sensing approach	AI^a^ and ML^b^ technology used	Clinical aim of AI and ML technology
Buckley et al [74], 2019	Accelerometer, gyroscope, and magnetometer	Inertial measurement unit	Head, lumbar region, and pelvis	Upper body acceleration and spatiotemporal characteristics	Wi-Fi	Data server	Opportunistic	Receiver operating characteristic and area under the curve	Determine the correlation of clinical feature and gait analysis
Coates et al [75], 2020	Accelerometer, light sensor, and temperature sensor	Smart band	Lumbar region	Sample entropy and gait	USB cable	Data server	Opportunistic	IAAFT^c^	Determine the correlation of clinical features
De Vos et al [76], 2020	Accelerometer, gyroscope, and magnetometer	Inertial measurement unit	Lumbar region, wrist, and feet	Gait and postural sway	Wi-Fi	Data server	Opportunistic	LASSO^d^ and random forest	Parkinson disease classification
Dominey et al [77], 2020	Accelerometer	Smartwatch	Wrist	Bradykinesia, dyskinesia, and sleep disturbance	Wi-Fi	Data server	Opportunistic	No	—^e^
Dunne-Willows et al [78], 2019	Accelerometer, light sensor, and temperature sensor	Smart band	Lumbar region	Gait	USB cable	Data server	Opportunistic	Phase algorithm	Gait analysis
Huo et al [69], 2020	Force sensor, accelerometer, gyroscope, magnetometer, and mechanomyography sensor	Smart band	Arm	Rigidity, tremor, and bradykinesia	Bluetooth	Data server	Opportunistic	1-NN^f^, AdaBoost classifier, and MLP^g^ neural networks classifier	Ranking Parkinson disease symptoms
Lacy et al [79], 2018	Electromagnetic sensor	Smart finger	Finger	Finger tapping	Sensor cable	Data server	Opportunistic	Echo state network	Parkinson disease classification
Lones et al [80], 2017	Accelerometer, gyroscope	Smart band	Head, chest, arms, and legs	Dyskinesia	Wi-Fi	Mobile phone	Opportunistic	IRCGP^h^	Parkinson disease classification
Mc Ardle et al [72], 2020	Accelerometer, light sensor, and temperature sensor	Smart band	Lumbar region	Gait	USB cable	Data server	Opportunistic	Black box	Parkinson disease classification
Mc Ardle et al [73], 2021	Accelerometer, light sensor, and temperature sensor	Smart band	Lumbar region	Gait and balance impairment	USB cable	Data server	Opportunistic	Area under the curve	Parkinson disease classification
Pantall et al [81], 2018	Accelerometer, light sensor, and temperature sensor	Smart band	Lumbar region	Sample entropy	USB cable	Data server	Opportunistic	Area under the curve	Parkinson disease classification
Pantall et al [82], 2018	Accelerometer, light sensor, and temperature sensor	Smart band	Unreported	Balance impairment and posture	USB cable	Data server	Opportunistic	Black box	Determine the correlation of clinical features
Payne et al [70], 2020	Accelerometer, gyroscope, and magnetometer	Inertial measurement unit and Smart band	Head, upper back, lumbar region, and ankles	Motor impairment and gait	Wi-Fi	Data server	Opportunistic	Area under the curve	Determine the correlation of clinical features
Rehman et al [83], 2019	Accelerometer, light sensor, and temperature sensor	Smart band	Lumbar region	Gait	USB cable	Data server	Opportunistic	Support vector machine and random forest	Gait analysis and Parkinson disease classification
Rehman et al [84], 2020	Accelerometer, light sensor, temperature sensor	Smart band	Lumbar region	Gait	USB cable	Data server	Opportunistic	PLS-DA^i^	Gait analysis and Parkinson disease classification
Rehman et al [85], 2020	Accelerometer, gyroscope, and magnetometer	Inertial measurement unit	Head, neck, lumbar region, and ankles	Gait	Wi-Fi	Data server	Opportunistic	PLS-DA	Gait analysis Parkinson disease classification
Stack et al [86], 2018	Accelerometer and gyroscope	Smart band	Wrist	Instability	Unreported	Unreported	Opportunistic	No	—
Stuart et al [87], 2015	Accelerometer, light sensor, temperature sensor, and eye tracker	Smart band and Smart glasses	Head	Eye movements and gait	USB cable and Wi-Fi	Data server	Opportunistic	No	—
van Wamelen et al [88], 2019	Accelerometer	Smartwatch	Wrist	Bradykinesia, dyskinesia, and levodopa equivalent dose	Wi-Fi	Data server	Opportunistic	Black box	Determine the correlation of clinical features
van Wamelen et al [89], 2021	Accelerometer	Smartwatch	Wrist	Rigidity, tremor, and bradykinesia	Wi-Fi	Data server	Opportunistic	Black box	Determine the correlation of clinical features

^a^AI: artificial intelligence.

^b^ML: machine learning.

^c^IAAFT: iterated amplitude–adjusted Fourier transform.

^d^LASSO: least absolute shrinkage and selection operator.

^e^Not available.

^f^NN: nearest neighbor.

^g^MLP: multilayer perceptron.

^h^IRCGP: implicit context representation Cartesian genetic programming.

^i^PLS-DA: partial least square discriminant analysis.

## Discussion

### Principal Findings

From the studies included in the scoping review, it was found that the main interest of wearable devices was the ability to provide more objective quantitative data compared with more subjective assessments such as the UPDRS in PD assessment [15,69]. The level and variety of data collected from wearable devices also provided more detailed information during the early diagnosis and long-term assessment of patients with PD. Furthermore, wearable devices provided continuous information that was believed to be more representative of the patient’s experience and enable more personalized care based on the data collected [70,77,83-85]. The overwhelming majority of the studies (19/20, 95%) used wearable devices that contained accelerometers and were placed at the lumbar region (11/19, 58%). This could be because many of the included studies used wearable devices to examine PD motor symptoms such as gait, bradykinesia, and dyskinesia [69,70,72,73,75-78,80,83-85,​87-89]. Accelerometers were found to be commonly used to measure physical activity, as evidenced by the systematic review conducted by Chan et al [95]. Of the studies that used accelerometers, 42% (8/19) used the AX3 manufactured by Axivity. The systematic review by Godhino et al [92] highlighted that the AX3 could reliably and accurately measure 3D accelerations from the lumbar region and could be used to support measurement in interventional assessments, as well as disease classification and grading. It was also found that users found the AX3 more practical to wear for longer periods compared with other wearable devices, and the positive user experience may have improved participant compliance [96]. These characteristics could explain why the Axivity AX3 was commonly used in the included publications, and it provided reliable and accurate measurements when conducting physical assessments while being comfortable and practical for use in a hospital setting. All the publications used an opportunistic sensing approach. This could be explained by the fact that opportunistic sensing can efficiently collect data about users’ activities and behaviors while minimizing user bias that may impact readings [97]. An opportunistic sensing approach is commonly used with body-worn sensors due to its ability to quantify physical characteristics while simultaneously providing different descriptive parameters such as data accuracy and sensor location [97]. As the included studies often monitored PD symptoms using body-worn sensors, an opportunistic sensing approach using accelerometers may be the most viable option to easily measure the variety of physical characteristics and user behaviors during the assessment period [69,72,73,75,77,​78,81-85,88,89].

In the previous systematic review by Rovini et al [34], Perumal and Sankar demonstrated that gait characteristics such as lower limb movement could be used to distinguish between patients with PD and healthy controls [98]. However, recent research has focused on obtaining more detailed information on PD spatiotemporal characteristics and determining the correlation between these characteristics to identify which biomarkers are the most beneficial for improving PD assessment. De Vos et al [76] and Mc Ardle et al [72] found that gait analysis continued to be beneficial. and characteristics including step length, step velocity, and turn duration could be used to successfully distinguish between patients with PD and those with other neurodegenerative conditions such as PSP, AD, and dementia with Lewy bodies (DLB), which can often present in a manner similar to PD. De Vos [76] et al identified parameters that were more significant in distinguishing patients with PD from those with other neurodegenerative conditions. Previous studies did not investigate which parameters were the most significant contributors to classifying PD or which appropriate parameters were the most appropriate to use. Although previous studies had identified that postural sway could be used as a biomarker for some PD subtypes such as prodromal PD [34], De Vos et al [76] identified that postural sway was less useful for discriminating between PSP and PD. Mc Ardle et al [72] conducted a study to determine if balance impairment markers such as jerk could be useful for differential PD assessments, but they found that the use of wearable devices to analyze jerk was limited. Although static eyes-open assessment using accelerometers could be used to accurately distinguish between patients with PD dementia (PDD), patients with AD, and healthy age-matched controls, balance impairment assessment was unable to identify significant differences among the patients with PDD, AD, and DLB once adjustments were made [73]. These results contrast with previous research that was able to find significant differences among the patients with PDD, AD, and DLB [99]. However, observations by Pantall et al [82] also showed that patients with PD experienced a decrease in postural jerk over time; however, it remains uncertain whether jerk increases or decreases over time and to what extent the results are due to age-related decline or PD progression. Although this study may have contradicted previous results, wearable devices may also have some benefit in providing more objective data regarding which differential assessments may be more appropriate depending on which medical conditions are being assessed. However, as the size and weight of wearable devices have decreased, studies have included more analyses of upper body movements using IMUs. Buckley et al [74] found that PD assessment by wearable devices could still be improved by including upper body analysis. The study identified that including upper body data analysis enabled more detailed information about gait characteristics such as swing time, asymmetry, and step length, which led to significant improvements when classifying PD gait compared with lower limb analysis alone. The studies included in the scoping review also attempted to make PD assessment more convenient by using wearable devices to reduce the need for specialist assessment facilities. Dunne-Willows et al [78] demonstrated that wearable device data combined with phase plots could be used as an alternative method to distinguish between patients with PD and healthy controls. It was consistently shown that patients with PD and healthy controls had phase plot characteristics for single-line, thin-line double, and oblique wing–phase plots. Different phase plots could also be used to identify particular gait characteristics in question to build a more complete and unique profile of the patient’s condition. It was found that thin-line double-phase plots demonstrated significant increases in step length and velocity when compared with parallel wing–phase plots [78]. Therefore, the novel use of phase plots could be used as a supplementary assessment tool to easily and conveniently produce an overview of gait in patients with PD.

Studies in the United Kingdom have continued to highlight the hardships of patients with PD and carers due to the increasing complexity of daily routines such as medication management and environmental risks in home settings, which can increase the risk of dangerous falls. The additional strain of care can have negative impacts on well-being, as patients and carers can feel unsupported; moreover, there are difficulties in carers and patients communicating their situation and symptoms to health care professionals [100]. Wearable devices can be a possible solution to collecting and providing detailed information on the patient’s PD status and symptoms, which can lessen the strain on patients and carers, as this can decrease the anxiety around whether the information is being relayed clearly to health care professionals. Current studies have shown that wearable devices can provide information on how medication treatment affects patient outcomes [70,76,82]. Newer studies included in the scoping review have shown development in addressing whether patients were receiving appropriate medication doses or identifying if the patient’s PD was controlled by using PKGs to identify novel biomarkers and correlations that may influence the patient’s condition [88,101]; for example, wearing-off [102] in levodopa therapy was identified by PKGs as a key clinical concern, allowing clinicians to modify medication or therapy to address wearing-off and facilitate discussion of new clinical findings with patients. PKGs were shown to be an appropriate support tool for minimizing incidents of undetected symptoms in newly diagnosed patients with PD, undertreated patients, and clinical bias, as well as for analyzing how symptoms progressed throughout the day [77,89]. The medication reminders provided by the PKGs were found to be valued by 80% of patients, enhancing self-management, and it was found that wearable devices provided additional data that patients found difficult to relay to the clinical team [77]. Dominey et al [77] reported that 97% (57/59) of patients with PD were willing to continue to use the PKG to support the management of their PD care and the belief that wearable device information represented their lived experiences. However, the data showed that clinicians needed to consider how information was presented to and understood by patients, as there were low levels of satisfaction with how PD information was presented. In a survey of the methods used to communicate information about the patients’ PD status, only 50% of surveyed patients were satisfied with phone calls, 47% with letters, 44% with clinician reports, and 27% with PKG graphs [77]. The main reported concerns were related to potential technical problems such as delays in receiving the information or whether the PKG accurately recorded information about their condition [77]. Technological literacy can often be a barrier to the effective adoption of wearable devices. Studies have found that older patients are less likely to be technologically literate than younger patients, and those from low-income backgrounds are less technologically literate than those from higher-income backgrounds [103]. The lower levels of satisfaction of the surveyed communication levels observed by Dominey et al [77] may potentially be due to lower levels of technological literacy among the older study participants. Therefore, it may be beneficial if triggered contacts are implemented to notify patients and clinicians of changes that require follow-up action and support channels where patients could have questions about the PKG answered. A study by van Wamelen et al [88] was able to analyze the relationships between different PD symptoms using regression analysis in which bradykinesia scores were associated with a low levodopa equivalent dose, and dyskinesia was related to a high levodopa equivalent daily dose. PKGs also identified that higher bradykinesia scores were correlated with gastrointestinal problems; however, PD-related gastrointestinal problems can be undiscussed, as patients are unaware that they are related to PD [88]. Therefore, using PKGs, clinicians can proactively discuss nonmotor symptom concerns with the patients if higher bradykinesia scores are observed over time. van Wamelen et al [89] demonstrated that newer wearables such as the PKG were able to provide more detailed information when PD symptoms fluctuated throughout the day where the time intervals could be easily divided into 3-h periods. Furthermore, the study investigated whether PD symptoms were affected by time variations in patients with early-stage PD, which remained relatively unexplored [89]. It was identified that motor assessments such as bradykinesia scores varied depending on the time of assessment, and UPDRS scores worsened throughout the day [89]. The common concern around current PD assessment scores is that the scores are too subjective and prone to variability [15]. However, van Wamelen et al [89] have shown that PD assessments can become more precise by using wearable devices such as the PKG to provide a baseline reading to account for changes in symptom severity based on the time of assessment [89]. The study showed that wearable devices could provide more personalized assessments and treatments because they collect more detailed data, particularly for patients diagnosed with early-stage PD [89]. Huo et al [69] further demonstrated the use of this information. By using wearable device data combined with ML algorithms, Huo et al [69] could quantify PD motor symptoms and classify the impact of PD motor symptoms to assess the patient’s severity of PD. Consequently, newer studies have shown that wearable devices can identify promising biomarkers that could be used to provide more objective quantitative data, as well as provide more information on how PD clinical features are correlated with each other. Studies also attempted to reduce the risk of falls for patients with PD. Falls are often a common cause of injury or death in older adults and can further negatively impact patient independence and quality of life due to further fear of falling [104]. Patients with PD are also susceptible to increased falls that incur unnecessary costs due to additional medical treatment or hospital admissions [105]. Costs are attributed to the medication received during inpatient care or purchased at local pharmacies and medical equipment such as walkers or braces [106]. However, in current research, wearable devices have identified stride time, and postural sway analysis could help support the early identification of patients with PD who are at a higher risk of falling, enabling support measures to be introduced earlier and prolonging independent living [75,82]. Furthermore, AI and ML developments combined with the use of wearable devices could be used to predict the risk of falls in patients with PD based on detecting subtle changes in instability among patients who were at risk of falling and could help capture more detailed data than video assessments alone [86]. These developments have beneficial applications in both the hospital and community settings. The data gathered by Stack et al [86] from wearable devices in a hospital laboratory setting were subsequently used to develop an algorithm to predict the risk of falls in patients with PD, and more research has been conducted on predictive algorithms related to patient falls in community settings [107]. For hospital assessments, predictive fall algorithms can provide more granular and personalized information when conducting mobility assessments such as TUG and chair transfer tests, which can support clinical decision-making [86]. In addition, the successful implementation of predictive fall algorithms may improve individualized care and minimize risks to patients when alone in health care environments such as nursing homes [107]. Therefore, current studies demonstrating the use of commercial PD wearable devices such as the PKG may not only have multiple applications by providing more information and increasing opportunities during hospital appointments but also provide more support for patients in home settings when away from the hospital.

AI and ML have been continuously incorporated and used to analyze wearable device data. However, accurately classifying PD characteristics can be challenging; therefore, Rehman et al [83] attempted to investigate how different assessment methods and equipment affected accuracy and ML models. Both continuous and intermittent walking methods were tested using the Axivity AX3 accelerometer and the GAITRite mat. The GAITRite mat is established as one of the gold standard devices for gait analysis [83]. Both the support vector machine and random forest ML models found that continuous walking performed better than intermittent walking with both the Axivity AX3 and GAITRite; however, the continuous walking method and the Axivity AX3 wearable accelerometer produced the highest levels of accuracy [83]. The results observed by Rehman et al [83] highlighted that the direct comparison of ML models using different assessment methods was limited and that further research should be conducted on standardized assessment methods that were not considered in the studies included in the previous systematic review by Rovini et al [34]. Therefore, the type of assessment method and equipment used can lead to varied results and levels of accuracy, and the combination of assessment methods and equipment used should be more extensively considered. Wearable devices in combination with AI and ML may also have the ability to develop more objective diagnosis and assessment criteria, although there has been little discussion about the use of AI and ML in PD assessment in previous research [34]. Current assessment methods such as the Hoehn and Yar scale can also be too simplistic to describe PD progression and provide a rank order of PD symptoms [108]. Assessment scores are also subjective [71]; therefore, disagreements on the assessment methods and scores are common [109-111]. However, more recent research by Stack et al [86] highlighted that the more subjective video reviews agreed with the wearable sensor data, suggesting that video review and wearable device use may still have beneficial applications in some PD assessments such as assessing the risk of falls. With the support of AI and ML algorithms, wearable devices could provide more objective assessment scores. Although previous attempts have used artificial neural networks to measure PD symptoms such as tremors [112], current research has innovated upon previous developments. It was found that computer voting classification algorithms based on the data collected by wearable devices were able to rank the most significant disease contributors for each PD symptom to calculate a weighted score to analyze the level of PD severity [69]. Huo et al [69] built upon previous research and were able to determine which symptoms contributed the most to the final UPDRS score while still being able to measure the fundamental symptoms (tremor, bradykinesia, and rigidity) that are often assessed. Echo state networks (ESNs) have also been used to classify PD. ESNs are distinctly different from artificial neural networks because of their ability to function nonlinearly, making them more suited to time-series analysis such as analyzing PD progression over time [113]. ESNs were found to be able to successfully diagnose PD at an accuracy rate similar to other algorithms that required more complex and substantial training [79,114]. It was found that the main advantage of using ESNs compared with other neural networks was the speed at which ESNs could be trained despite the inclusion of many different parameters, making ESNs suitable for interpreting complex information and patterns [79].

It was found that the specificity and sensitivity of wearable devices were good overall but still could be improved and were influenced by where the wearable device was placed on the body. Using the Opal wearable devices, Buckley et al [74] showed that wearable devices were able to discriminate between patients with PD and healthy age-matched controls for 62% (10/16) of the measured spatiotemporal variables and 75% (37/49) of the measured upper body variables, which were validated via receiver operating characteristic curve analysis with a *P* value of <.05. When gait variables were entered in a forward stepwise fashion, all models showed a good ability to discriminate between patients with PD and healthy age-matched controls, with AUC values of ≥0.88. The inclusion of upper body variables such as measurement of the smoothness of head movement in the spatiotemporal model led to a significant improvement; however, the AUC values only increased by 0.01 to 0.02 [74]. The novel inclusion of upper body analysis in gait analysis highlighted that not all PD gait data could be captured by spatiotemporal analysis, and the inclusion of upper body acceleration analysis provided additional details. The investigation also highlighted that upper body acceleration was not significantly correlated with postural control, and findings may further support that head movement and gait require different targeted therapies [115]. Rehman et al [85] also found that a combination of lower and upper body acceleration measurements using IMUs could distinguish between patients with PD and healthy controls. However, Rehman et al [85] used a turning algorithm that had not been explored in previous research [34]. Exploring a combination of IMU locations using a single sensor and the partial least square discriminant analysis classifier resulted in high levels of specificity, ranging from 84% to 92%. The highest levels of accuracy were found when multiple sensors were used and placed on the neck, lumbar region, head, and inner ankle, which led to 98% accuracy and 100% specificity, with the IMU data validated by video by 2 independent reviewers. The validation of wearable device data often relies on the established gold standard PD assessment methods. Wearable device validation involved motion capture systems or videos that were then reviewed by specialist clinicians with experience in assessing patients with PD [80,85,86]. Other methods have validated wearable devices with known gold standard PD assessments such as the UPDRS scale [69,70,82,87-89] or equipment such as the GAITRite mat sensor [75,83,85]. Some studies used algorithms that had been validated in other similar neurodegenerative conditions or in older adults [72]. Technical validation of the data was completed mainly by the use of statistical methods including 10-fold cross-validation, ANOVA, and the Shapiro-Wilkes test [73,76,79]. Although video validation by independent reviewers is a common method in PD assessment, the reliability of video validation remains inconclusive and needs further work to determine if it is an appropriate form of validation [116]. Some studies reported that video assessment was not a reliable form of validation, and the variability of assessments was a cause for concern [117]. In contrast, other studies believed that video assessment was a suitable method for validating PD diagnoses [116,118].

One of the main challenges limiting the ability to support the effectiveness of wearable devices and their widespread implementation in PD care is the lack of medical-grade regulations of wearable devices currently being used. Currently, the official regulation of wearable devices continues to be a challenge in the United Kingdom. Less than half of the studies (9/20, 45%) used a wearable device that was approved by official regulatory bodies. The lack of medical-grade certification for many wearable devices used in the studies included in the scoping review could subsequently increase the uncertainty around the validity of the data and the results. Only wearable devices manufactured by APDM (Ambulatory Parkinson’s Disease Monitoring), Global Kinetics, ClearSky, and McRoberts had medical certification, but the certification was approved by regulatory bodies outside the United Kingdom [70,74,76,77,79,80,85,88,89]. The wearable devices were only clinically approved by the United States regulatory body, the FDA, or the European Union regulatory body via the CE medical mark. Although changes in United Kingdom health care regulations remain unclear, eventually, wearable devices that will be used in United Kingdom clinical care will require approval by the Medicine and Healthcare products Regulatory Agency and will not be covered under the CE medical mark [119]. Therefore, implementing wearable devices in routine PD care based on the evidence from current studies may prove to be challenging. The challenges around wearable device approval are evident in countries such as Australia that uses the Therapeutic Goods Administration (TGA) to regulate approved wearable devices aimed for clinical use. Only a few wearable devices have been approved by the TGA to monitor vital signs and physiological features in a clinical setting and were manufactured by Masimo Corporation, The Waringa Group, and GE Healthcare [120-122]. Particularly in the United Kingdom, there is a severe lack of medical device and data security regulations or guidance, which has been exacerbated by the recent changes in the UK political landscape [94,123]. Currently, there is little regulation or guidance on whether wearable devices should be approved for clinical use in the United Kingdom. Only some wearable devices such as the PKG have been approved by non-UK regulatory bodies and are unofficially recommended by NICE in the UK [77,88,89,124]. Most recently, the UK committee NICE has provided conditional recommendations for the use of 5 wearable devices in UK PD care, including the PKG [94]. Furthermore, 1 NHS government proposal has been planned to issue PKGs to >120,000 people living with PD in the United Kingdom, which may provide more robust evidence of interventional scalability [124]. However, the results of this initiative have yet to be published, and there is no official guidance on whether recommended wearable devices such as the PKG can be prescribed [94,124]. Consequently, the lack of medical-grade approved wearable devices and limited research on these NICE-recommended devices within the United Kingdom has generated uncertainty regarding the accuracy and reliability of data from recent studies [94].

### Comparisons With Prior Work

Previously, systematic reviews provided only a brief overview of the current applications of wearable devices used in PD care [34]. This scoping review builds upon previous systematic reviews by providing an updated commentary with more details on how wearable devices and wearable device data are being used and the current challenges of implementing wearable devices in mainstream PD care. Providing an updated review is important due to the speed at which technological development occurs [59]. The scoping review details the common device brands and wearable device technologies that are currently being used by health care professionals, as well as how these wearable devices are commonly applied in PD care. Furthermore, previous systematic reviews only focused on providing an overview of wearable device use, with little discussion about the potential challenges related to the use of PD wearable devices. For example, previous studies hypothesized how AI and ML might use wearable device data, but there was insufficient detail on precisely how AI and ML could be used to support PD assessment [34]. In addition, the systematic review did not sufficiently document how wearable device placement could impact the observed results [34].

The use of wearable devices in clinical care requires vigorous regulation and guidance. Currently, the practicalities and challenges of regulating and implementing wearable devices in PD care remain relatively unexplored, with very few wearable devices being approved by regulatory bodies for clinical use. Most wearable devices approved for clinical use are related to vital sign monitoring and are approved by regulatory bodies such as the TGA in Australia and the US FDA [77,120-122]. However, this review explores how uncertainty in regulation could restrict the ability to implement wearable devices in health care, which has not been analyzed in previous studies [34]. Recent political changes in the United Kingdom, for example, due to Brexit, have resulted in planned changes to UK health care regulations, which are expected to deviate from the previously shared European Union CE medical mark from 2023 onward [119]. Currently, it is more uncertain which wearable devices can be approved for clinical use and implemented in wider UK PD care. Therefore, this scoping review highlighted a challenge unique to the United Kingdom. Consequently, the scoping review is an important record of how ongoing regulatory changes may impact wearable device implementation related to clinical practice in areas such as UK PD care.

The current lack of studies analyzing wearable device security risks was also identified as a significant research gap [47,72,75,80]. In a recent scoping review by Huhn et al [125], many studies did not consider data accessibility, sharing and security. This observation is similar to that of the studies included in this scoping review, as only Dominey et al [77] documented the challenges of obtaining suitable data storage and management systems to store the increasingly large amounts of data collected during the study. Furthermore, only Mc Ardle et al [72] and Payne et al [70] considered the type of computer application used to store the wearable device data securely. The platforms used were e-Science Central, which allowed secure storage and sharing of data using workflow editors and access control lists [126] or held in a centralized database where access was regulated by named user access and encrypted passwords [70]. Currently, the different data policies that countries must abide by can make accessing data difficult. However, in the United Kingdom, how General Data Protection Regulation continues to be implemented is uncertain and could potentially make wearable device implementation in UK health care challenging [123]. Moreover, security mechanisms for wearable devices are left to the manufacturers with no clear legal obligations, and IT security is often insufficiently assessed [46,47,127,128]. These proposed frameworks have previously been criticized as unsuitable for medical devices, and approved devices continue to be susceptible to cyberattacks [47]. Medical device regulatory bodies such as the FDA have limited classification and criteria relating to wearable device safety and security, as the devices are not rigorously assessed for IT security, which has led to product recalls due to incorrect risk classification [46,47].

This scoping review also identified that there was a lack of age diversity in study participants with PD, as most participants were aged >65 years. Older patients with late-onset PD (LOPD) would often have more severe PD and more distinctive symptoms [129]. Younger patients with young-onset PD (YOPD) also face different challenges due to their stage in life such as marital challenges and uncertainty around employment, particularly as these patients are supposed to be in the prime of their lives [129]. It is uncertain how wearable devices could be optimized for patients with YOPD or how the current results would differ for patients with YOPD. The age range of participants in the included studies ranged from 58.0 to 78.0 years [69,72-79,81-86,88,89]. The lack of research on wearable device use in younger patients with PD is likely because PD is not as prevalent in people aged between 21 and 40 years [129]. Importantly, differential diagnoses of patients with YOPD can differ because clinicians conducting the PD assessments are likely to assume that the patient has a different disease that may share similar symptoms with PD such as mitochondrial disease or drug-induced parkinsonism [129]. Compared with older patients with PD who often present with clinical features such as gait instability, which the scoping review identified as a common symptom analyzed using wearable devices [83-85], younger patients with YOPD can more often present with rigidity and cramping, and it was found that dystonia was significantly featured in patients with YOPD [129,130]. It has been proposed that the observed differences related to dystonia symptoms are related to the amount of degradation of the caudate and putamen within the brain, as it was observed that patients with YOPD had higher amounts of caudate than putamen, whereas patients with LOPD had similar levels of caudate and putamen [131]. As for nonmotor symptoms, patients with YOPD are more likely to experience signs of depression that is likely due to the declining quality of life at a relatively young age [130]. Due to the differences in clinical presentations between patients with YOPD and LOPD, as well as the different challenges these demographics face, more research is required on how wearable device use differs in patients with YOPD and can improve clinical outcomes for younger patients with PD. The lack of diversity in the age range of study participants with PD may be partly explained by different UK hospitals having different research interests, which would influence the type of studies that are published. Furthermore, variations in geographic location may affect the ability to recruit and include certain patient demographics. In 55% (11/20) of the included studies, the research was conducted in Newcastle hospitals that form part of the Newcastle upon Tyne Hospitals NHS Foundation Trust [132]. The trust is funded by the National Institute of Health Research, which has made investments in clinical research and facilities such as the Clinical Ageing Research Unit (CARU) that has enabled hospitals in Newcastle to be at the forefront of PD research [132]. Current research at the CARU heavily involves research on PD and is equipped with specialized facilities such as the Human Movement Laboratory to investigate gait and mobility [132]. Many leading experts are also associated with the CARU and specialize in neurological disorders such as PD and gait impairment in older patients [132]. Consequently, this may reveal the reason most PD secondary care research in the United Kingdom was conducted at Newcastle upon Tyne Hospitals NHS Foundation Trust: access to leading PD experts and specialized facilities, resulting in more PD studies involving patients aged >65 years.

To investigate a wider variety of demographics of patients with PD, scale wearable device clinical trials to obtain more robust evidence to obtain medical-grade approval, and determine the additional costs related to wearable device implementation, further cost-effectiveness analysis is needed. Currently, there is a lack of analysis regarding the cost-effectiveness of wearable devices in PD care. Only a few studies (3/20, 15%) included in this scoping review provided a brief commentary on the cost-saving potential of wearable devices in PD care [72,75,80]. Importantly, the studies did not provide any analysis of the claims that using accelerometers in PD assessment such as gait analysis would be a cost-effective alternative compared with the established specialized gait laboratories and how an estimated US $110 million saved per year in England could be achieved by using wearable devices in PD care [72,75,80]. Widespread technological implementations also require additional investment into data storage and management software as discovered by Dominey et al [77], as well as the overhead costs of computing if ML and AI are used in combination with wearable devices [79,133]. Furthermore, wearable devices require repair and upkeep, but inventory and procurement costs can vary widely [133]. Munoz et al [134] analyzed the cost-effectiveness of nonwearable devices in the early detection of PD in the United States, and the analysis may provide some evidence that wearable devices could become cost-effective. It was found that PD technology may be quite cost-effective, as the lives of patients with early-stage diagnosed PD could be improved by up to 0.33 quality-adjusted life years (QALYs) estimated at US $31,305 per QALY, which was slightly over the limit of US $25,000 per QALY for the treatment to be considered very effective [134]. However, how cost-effectiveness translates to wearable devices used in PD care in the UK health care system remains unknown.

### Limitations

Because of the unique changes to the UK political and regulatory landscape, changes in UK regulations have occurred. Therefore, the scoping review focused on PD studies in the United Kingdom because of the current uncertainty and changes in UK clinical and technological regulations [48,119,123]. However, the limited scope may have increased the chance of publication bias, as the scope was more focused [58].

The publication search was conducted only by the first author. Although the inclusion of eligible studies and the search strategy methodology were supervised by the project supervisor and corresponding author, studies may have been missed or there may have been some variation during the screening process. In addition, the search strategy was limited to a few of the main established peer-reviewed databases, and peer-reviewed publications were reviewed only if they were written in English. Therefore, the identified publications may not be completely representative of the research available, as contributions made by technologically advanced far eastern countries such as South Korea, China, and Japan [131,135,136] were excluded. Gray literature was not included as the quality of information could vary greatly, so it was decided to search only established peer-reviewed databases to ensure a minimum standard of quality and credibility of the publications included [64,65]. Furthermore, searching gray literature is time-consuming and extensive and may not yield further benefits due to the rapid developments of technological advancements and sources that are potentially inaccessible in the future [65]. Therefore, we decided to follow the recommendations of Cochrane to focus on established peer-reviewed databases [66]. Conference abstracts, reviews, reports, and editorials were also excluded from this scoping review because these sources were often inadequately recorded, contained unsuitable information to enable sufficient data extraction of the study characteristics of interest, or were not published in full. By contrast, a study protocol is a structured and formal document that provides comprehensive information on the methodology and justification of the value of the research related to the topic of interest in the scoping review. The concerns around including specific publication formats were reflected in previous research. Hackenbrioch et al [58] explored whether certain sources such as conference abstracts were appropriate for inclusion in systematic reviews and found that conference abstracts contained insufficient information in areas such as the study details needed for data extraction, eligibility criteria, and contacts to acquire further study information.

The study limitations were mainly due to the lack of wearable device regulations in the United Kingdom. Therefore, the accuracy of the results remains uncertain because the devices have yet to be officially approved as medical-grade devices that are suitable for use in clinical care in the United Kingdom [124]. Furthermore, the number of participants within each included study was often relatively small, with most studies having ≤100 participants [69,70,72,75,76,78,80,82,85-87]. The low number of participants may have been due to challenges in recruiting participants with PD [51]. Furthermore, study participants with PD tended to be older, and there is little information on how wearable devices could benefit patients with YOPD and how the different challenges faced by patients with YOPD can impact the results [129,130]. Although there have been proposals to introduce wearable devices to the wider demographics of patients with PD to gather larger volumes of data, additional studies are still required to understand if the currently observed results translate to larger study cohorts. Concerns regarding study overlap were also identified because Newcastle Hospital conducts extensive PD research with dedicated PD clinics, which may have resulted in selection bias or study participants participating in multiple studies at Newcastle Hospital [132]. Study participant overlap and selection bias may have affected characteristics such as disease severity, disease duration, and outcomes observed within study participants with PD across the Newcastle Hospital studies where results may not be reflective of the general population with PD [137]. In addition, the confirmation of whether the study participants had overlapped in any of the studies taking place in Newcastle Hospital could not be obtained from the authors.

### Conclusions

This scoping review provides an overview of the common types and brands of wearable devices currently used in PD studies in the United Kingdom. The promising benefit of using wearable devices is the ability to provide more detailed information when analyzing PD symptoms and how each clinical characteristic affects the presentation of PD symptoms. Furthermore, the availability of quantitative data combined with AI and ML to determine the most significant variables may improve or replace current but subjective assessment tools such as the UPDRS. These benefits have the potential to improve PD diagnosis due to greater levels of information to support assessments, allowing clinicians to improve the early diagnosis of PD or differentiate it from other neurodegenerative diseases. Due to the ease of use of wearable devices, this may reduce reliance on costly specialist assessment centers that are only available at specific hospitals. Furthermore, the wearable devices are able to continuously monitor the patients’ symptoms and provide a more accurate representation of the patient’s experience, especially as it was found that the severity of PD symptoms can change throughout the day. This has benefits not only in hospital settings but also in the community because clinicians can be more easily alerted to when treatment is ineffective and personalize treatment plans.

However, several areas need further research or need to be addressed before wearable device use in UK PD care can be confidently recommended. The main cause of uncertainty is the lack of UK-approved medical-grade use wearable devices. Recommended wearable devices such as the PKG have only been recommended on a conditional basis, and the guidance is potentially subject to change. In addition, the lack of large-scale studies and research on wearable device use in younger patients with PD fuels further uncertainty about the accuracy and reliability of the results. Technological innovation can also be costly and can introduce new challenges. Currently, the included studies contain a lack of analysis about the cost-effectiveness of using wearable devices in UK PD care such as the need to invest in data management systems and the cost of computing data. Furthermore, there is a lack of analysis on the cybersecurity risks of using wearable devices in PD care, as well as the challenges surrounding the lack of proper guidance and testing of wearable devices that are being implemented in health care.

If the outlined challenges can be overcome and further researched, wearable devices have huge potential to improve PD management, diagnosis, and assessment. However, several areas of research need to be addressed before individuals can be confident that the use of wearable devices is effective in UK PD care and that the current recommendations to implement wearable devices in clinical care are cost-effective.

## Appendix

Multimedia Appendix 1 PRISMA (Preferred Reporting Items for Systematic Reviews and Meta-Analyses) checklist.

Multimedia Appendix 2 Study protocol.

Multimedia Appendix 3 Search strategy.

Multimedia Appendix 4 Extracted features form.

## Abbreviations

AD: Alzheimer Disease
AI: artificial intelligence
APDM: Ambulatory Parkinson’s Disease Monitoring
AUC: area under the curve
CARU: Clinical Ageing Research Unit
CE: Conformité Européenne
DLB: dementia with Lewy bodies
ESN: echo state network
FDA: Food and Drug Administration
IMU: inertial measurement unit
LOPD: late-onset Parkinson disease
ML: machine learning
NHS: National Health Service
NICE: National Institute of Clinical Excellence
PD: Parkinson disease
PDD: Parkinson disease dementia
PICO: population, patient, or participants, interventions, comparators, and outcomes
PKG: Parkinson’s KinetiGraph
PRISMA-ScR: Preferred Reporting Items for Systematic Reviews and Meta-Analyses extension for Scoping Reviews
PSP: progressive supranuclear palsy
QALY: quality-adjusted life year
TGA: Therapeutic Goods Administration
TUG: timed up-and-go
UPDRS: Unified Parkinson’s Disease Rating Scale
YOPD: young-onset Parkinson disease
