# Supplementary material for: An Exploration of Wearable Device Features Used in UK Hospital Parkinson Disease Care: Scoping Review
Source: J Med Internet Res. 2023 Aug 18;25:e42950. doi: 10.2196/42950 (PMC10474516; doi:10.2196/42950)
Supplement: Multimedia Appendix 2 [file jmir_v25i1e42950_app2.docx]

**An exploration of wearable device features used in UK hospital Parkinson’s disease care: A scoping review protocol**

William Tam^a^; **Mohannad Alajlani^a^**; Alaa Abd-alrazaq^b^*

^a^ WMG, International Manufacturing Centre, University of Warwick, Coventry, United Kingdom

^b^ AI Center for Precision Health, Weill Cornell Medicine-Qatar, Doha, Qatar

^*^ Corresponding author.

Dr Alaa Abd-alrazaq

E-mail address: aaa4027@qatar-med.cornell.edu

# **Objective**

The objective of the scoping review was to explore the features of wearable devices used for Parkinson’s disease care in hospitals within the United Kingdom as reported in the literature. The scoping review aims to build upon previous knowledge to provide more detailed information on the clinical features of Parkinson’s disease currently being analysed by wearable devices, as well as the type of wearable devices and brands used in Parkinson’s disease care.

# **Methods**

Scoping review, PRISMA guidelines will followed to develop the scoping review.

# **Search strategy**

1. Search sources:

Electronic databases: PubMed/MEDLINE, Embase (via OVID) and Cochrane Library

1. Search terms:

Disease-related terms:

Population/disease:

Parkinson’s disease OR parkinsonisms OR bradykinesia OR dyskinesia OR gait OR “postural instability” OR Parkinson’s disease dementia OR “idiopathic parkinsonism” OR “paralysis agitans” OR “shaking palsy”

Intervention:

"wearable device" OR "smart watch" OR "smartwatch" OR accelerometer OR gyroscope OR "PKG" OR "Parkinson's KinetiGraph" OR "IMU" OR “inertial measurement unit” OR “inertial sensor” OR AX3 OR Opal* OR "LID-Monitor" OR "PD-Monitor" OR "Dynaport Movemonitor" OR "eye tracker" OR "smart glasses" OR "smart band" OR "smartband" OR Axivity OR APDM OR Dikablis* OR Ergoneers OR "ClearSky" OR "magnetometer sensor" OR "electromagnetic sensor" OR "mechanomyography sensor" OR "smart insole" OR "smart clothing" OR "smart bracelet" OR "headband" OR "head band" OR "smart shoe" OR "fitness tracker" OR Garmin OR Fitbit OR "Samsung Galaxy Fit" OR "Apple Watch"

AND

hospital OR “National Health Service” OR NHS OR “NHS Trust”

AND

“UK” OR “United Kingdom”

# **Eligibility criteria**

1. Population/Disorders:

*Inclusion:* People with confirmed Parkinson’s disease. No restrictions regarding their age, gender or ethnicity.

*Exclusion:* Animals, patients with other medical conditions and people with Parkinson’s disease undertaking care outside of the United Kingdom.

1. Intervention:

*Inclusion:* Wearable devices regardless of their purpose and use. Examples of wearable devices include smartwatches, VR headsets, smart glasses, smart bracelets etc.

*Exclusion:* Non-wearable devices such as portable devices (e.g. mobile phones and tablets).

1. Compartor:

No comparator is required (if comparators are available in the literature these may comparators such as health-age matched controls and baseline readings).

1. Outcome:

No restrictions applied.

1. Setting:

*Inclusion:* Parkinson’s disease research or care within hospitals or in specialised laboratories located on or within hospital grounds.

*Exclusion:* Parkinson’s care or research taken in the community (e.g. GP practice, community care homes or the patient’s home). Care or research taking place outside the United Kingdom.

1. Language of Study:

*Inclusion:* English.

*Exclusion:* Any other language.

1. Study design:

No restrictions applied.

1. Year of study:

2017-2022.

1. Country of publication:

No restrictions applied.

1. Type of publication:

*Inclusion:* Peer-reviewed articles, theses, dissertations, study protocols, full conference papers

*Exclusion:* Reviews, preprints, conference abstracts, editorials, commentaries, non-peer-reviewed articles

# **Study selection process**

Two step process: Initial screening of literature via the assessment of titles and abstracts followed by rading of full texts of literature that passed the initial screening.

# **Data extraction process**

1. Study features:

Year of publication, study location, hospital, publication type, sample size, male:female study participant ratio, mean age of participants.

1. Intervention features:

Device brand, manufacturer, wearable device status, wearable device aim, sensor, placement of wearable device, measured biosignal, connectivity, host device, sensing approach, artificial intelligence or machine learning technology used.

**Study and intervention features were subjected to change depending on the features reported in the literature and reviewer feedback.*

# **Data extraction for paper**

1. Year published
2. Study location
3. Hospital
4. Publication type
5. Sample size
6. Male:Female (Ratio of male to female study participants)
7. Mean age in years
8. Parkinson’s disease duration in years*
9. Parkinson’s disease severity*
10. Control*
11. Device brand (Name of device)
12. Manufacturer (Which company produces the device)
13. Wearable device status (Commercial or Non-commercial device)
14. Regulatory approval for clinical use*
15. Wearable device aim (The aim for using the wearable device e.g. monitoring, prediction etc.)
16. Sensor (The type of sensor used in the wearable device e.g. accelerometer, gyroscope, force sensor etc.)
17. Wearable device type (Type of wearable device e.g. smart band, smartwatch, inertial measurement unit)
18. Placement of wearable device (Where the wearable device was placed on the body e.g. head, neck, back etc.)
19. Measured biosignal (What clinical feature was measured by the wearable device e.g gait, bradykinesia, dyskinesia, eye movement etc.)
20. Connectivity (How was the wearable device connected e.g. via Wifi connection, USB cable, Bluetooth etc.)
21. Host device (The connected device used to store transferred wearable device data e.g. Data server, laptop, mobile phone etc.)
22. Sensing approach (Opportunistic vs. Participatory)
23. Length of time wearable device was used*
24. Artificial intelligence or machine learning technology used (E.g. Random forest, echo state networks, Support vector machine, blackbox etc.)
25. Clinical aim of Artifical Intelligence/Machine Learning technology*

| *Measured outcome | What is the outcome that the study measured?   1. What wearable device brands were used? 2. What was the aim of wearable device use? 3. How were wearable devices commonly used? 4. What sensors did wearable devices use? 5. What biosignals were measured using wearable devices? 6. Was artificial intelligence or machine learning used alongside wearble devices? |
| --- | --- |

# **Data analysis and synthesis**

Narrative synthesise of literature.

# **Results**

The literature search was planned to be undertaken around May to June 2022.

# **Discussion**

The scoping review would provide further information on the type of wearable devices and brands used in Parkinson’s disease care within hospitals throughout the United Kingdom. The changes in healthcare and clinical device regulations due to the recent political changes have resulted in a unique case that requires further investigation. Due to increased technological innovation, there has been continued interest in incorporating wearable devices into clinical care. Wearable devices have the potential to improve Parkinson’s disease care and assessment using quantitative analysis with the support of Artificial Intelligence and Machine Learning algorithms compared to more traditional and subjective assessment criteria. By collating current research researchers can determine how wearable devices are being used and the current limitations. Furthermore, the scoping review identifies promising research and current research gaps that require further investigation.

# **Esimate Time Table**

| Task/ Time | W1 | W2 | W3 | W4 | W5 | W6 | W7 | W8 | W9 | W10 | W11 | W12 | W13 | W14 | W15 | W15 | W17 | W18 | W19 | W20 | W21 |
| --- | --- | --- | --- | --- | --- | --- | --- | --- | --- | --- | --- | --- | --- | --- | --- | --- | --- | --- | --- | --- | --- |
| Writing the protocol |  |  |  |  |  |  |  |  |  |  |  |  |  |  |  |  |  |  |  |  |  |
| Searching databases |  |  |  |  |  |  |  |  |  |  |  |  |  |  |  |  |  |  |  |  |  |
| Study selection |  |  |  |  |  |  |  |  |  |  |  |  |  |  |  |  |  |  |  |  |  |
| Data extraction |  |  |  |  |  |  |  |  |  |  |  |  |  |  |  |  |  |  |  |  |  |
| Data synthesis |  |  |  |  |  |  |  |  |  |  |  |  |  |  |  |  |  |  |  |  |  |
| Writing up |  |  |  |  |  |  |  |  |  |  |  |  |  |  |  |  |  |  |  |  |  |

# **Author contributions**

The review topic was conceived by WT. The protocol was written by WT, with final revision by AA-a and MA.

# **Funding**

The review is not funded.
