# Supplementary material for: An Exploration of Wearable Device Features Used in UK Hospital Parkinson Disease Care: Scoping Review
Source: J Med Internet Res. 2023 Aug 18;25:e42950. doi: 10.2196/42950 (PMC10474516; doi:10.2196/42950)
Supplement: Multimedia Appendix 3 [file jmir_v25i1e42950_app3.docx]

**Appendix 2: Search strategy**

Database(s): **Embase**1996 to 2022 Week 24. Monday, June 6, 2022

| **#** | **Searches** | **Results** |
| --- | --- | --- |
| 1 | exp Parkinson’s disease/ | 40544 |
| 2 | parkinsonism.tw. | 77998 |
| 3 | bradykinesia.t.w. | 7101 |
| 4 | dyskinesia.t.w. | 97442 |
| 5 | gait.t.w. | 75184 |
| 6 | “postural instability”.t.w. | 5405 |
| 7 | “parkinson's disease dementia”.t.w. | 1005 |
| 8 | “idiopathic parkinsonism”.t.w. | 261 |
| 9 | “paralysis agitans”.t.w. | 1179 |
| 10 | “shaking palsy”.t.w. | 81 |
| 11 | exp wearable devices/ | 6483 |
| 12 | "smart watch".tw. | 272 |
| 13 | smartwatch.tw. | 591 |
| 14 | accelerometer.tw. | 17763 |
| 15 | gyroscope.tw. | 2438 |
| 16 | "inertial sensor".tw. | 1162 |
| 17 | "inertial measurement unit".tw. | 2341 |
| 18 | IMU.tw. | 3109 |
| 19 | PKG.tw. | 3164 |
| 20 | “Parkinson’s KinetiGraph” .tw. | 24 |
| 21 | AX3.tw. | 161 |
| 22 | Opal.tw. | 2318 |
| 23 | “LID-Monitor”.tw. | 0 |
| 24 | “PD-Monitor”.tw. | 4 |
| 25 | “Dynaport Movemonitor”.tw. | 20 |
| 26 | "eye tracker".tw. | 1401 |
| 27 | “smart glasses” | 257 |
| 28 | “smart band” | 30 |
| 29 | APDM.tw. | 158 |
| 30 | Axivity.tw. | 63 |
| 31 | Dikablis*.tw. | 5 |
| 32 | Ergoneers.tw. | 2 |
| 33 | "ClearSky".tw. | 166 |
| 34 | “magnetometer sensor".tw. | 14 |
| 35 | "electromagnetic sensor".tw. | 105 |
| 36 | "mechanomyography sensor".tw. | 63 |
| 37 | "smart clothing".tw. | 82 |
| 38 | "smart bracelet".tw. | 22 |
| 39 | "smart insole".tw. | 43 |
| 40 | headband.tw. | 302 |
| 41 | "head band".tw. | 26 |
| 42 | "smart shoe".tw. | 18 |
| 43 | "fitness tracker".tw. | 154 |
| 44 | Garmin.tw. | 280 |
| 45 | Fitbit.tw. | 1130 |
| 46 | "Samsung Galaxy Fit".tw. | 1 |
| 47 | "Apple Watch".tw. | 237 |
| 48 | hospital.tw. | 6329625 |
| 49 | 1 or 2 or 3 or 4 or 5 or 6 or 7 or 8 or 9 or 10 | 173857 |
| 50 | 11 or 12 or 13 or 14 or 15 or 16 or 17 or 18 or 19 or 20 or 21 or 22 or 23 or 24 or 25 or 26 or 27 or 28 or 29 or 30 or 31 or 32 or 33 or 34 or 35 or 36 or 37 or 38 or 39 or 40 or 41 or 42 or 43 or 44 or 45 or 46 or 47 | 36029 |
| 51 | 48 and 49 and 50 | 1261 |
| 52 | limit 51 to (english language and yr="2017 -Current") | 503 |
| 53 | limit 52 to exclude medline journals | 322 |

| **Database** | **Query** | **Results** |
| --- | --- | --- |
| **MEDLINE/PubMed** | ("parkinson's disease"[MeSH Terms]) OR (parkinsonism) OR (bradykinesia) OR (dyskinesia) OR (gait) OR ("postural instability") OR ("idiopathic parkinson's disease") OR ("paralysis agitans") OR ("shaking palsy") AND ("wearable device") OR ("smart watch") OR ("smartwatch")) OR (accelerometer) OR (gyroscope) OR ("PKG") OR ("Parkinson's KinetiGraph") OR ("IMU") OR (“inertial measurement unit”) OR (“inertial sensor”) OR (AX3) OR (Opal*) OR ("LID-Monitor") OR ("PD-Monitor") OR ("Dynaport Movemonitor") OR ("eye tracker") OR ("smart glasses") OR ("smart band") OR ("smartband") OR (Axivity) OR (APDM) OR (Dikablis*) OR (Ergoneers) OR ("ClearSky") OR ("magnetometer sensor") OR ("electromagnetic sensor") OR ("mechanomyography sensor") OR ("smart insole") OR ("smart clothing") OR ("smart bracelet") OR ("headband") OR ("head band") OR ("smart shoe") OR ("fitness tracker") OR (Garmin) OR (Fitbit) OR ("Samsung Galaxy Fit") OR ("Apple Watch") AND (hospital)  Publication Date: 5 years (search date: 6^th^ June 2022)  Language: English | 4175 |

Database(s): **Cochrane Library**

| **#** | **Searches** | **Results** |
| --- | --- | --- |
| 1 | MeSH descriptor: [Parkinson’s Disease] explode all trees | 4758 |
| 2 | (parkinsonism) OR (bradykinesia) OR (dyskinesia) OR (gait): ti, ab, kw | 14317 |
| 3 | #1 OR #2 | 8436 |
| 4 | (“postural instability”) OR (“paralysis agitans”) OR (“idiopathic Parkinson’s disease”) OR (“idiopathic parkinsonism”): ti, ab, kw | 1110 |
| 5 | #3 OR #4 | 8836 |
| 6 | (accelerometer) OR (gyroscope) OR (“smart watch”) OR (smartwatch) OR (PKG): ti, ab, kw | 3852 |
| 7 | (“wearable device”) OR (“Parkinson’s KinetiGraph”) OR (“IMU”) OR (“inertial measurement unit”) OR (“AX3) | 623 |
| 8 | (Axivity) OR (Opal) OR (“LID-Monitor”) OR (“PD-Monitor”) OR (“Dynaport Movemonitor”) | 346 |
| 9 | (“smart glasses”) OR (“smart band”) | 59 |
| 10 | (smartband) OR (Dikablis) OR (Ergoneers) OR (ClearSky) OR (“magnetometer sensor”) | 44 |
| 11 | (“electromagnetic sensor”) OR (“mechanomyography sensor”) OR (“smart insole”) OR (“smart bracelet”) | 42 |
| 12 | (headband) OR (“head band”) OR (“smart shoe”) OR (“fitness tracker”) OR (Garmin) | 400 |
| 13 | (“Samsung Fit”) OR (“Apple Watch”) | 59 |
| 14 | #6 OR #7 OR #8 OR #9 OR #10 OR #11 OR #12 OR 13 | 4189 |
| 15 | #5 AND #14 | 77 |
