# Supplementary material for: An Exploration of Wearable Device Features Used in UK Hospital Parkinson Disease Care: Scoping Review
Source: J Med Internet Res. 2023 Aug 18;25:e42950. doi: 10.2196/42950 (PMC10474516; doi:10.2196/42950)
Supplement: Multimedia Appendix 4 [file jmir_v25i1e42950_app4.docx]

**Appendix 3: Extracted Features Explanation**

| **Data extraction form** | | |
| --- | --- | --- |
| **Features Name** | **Definition** | **Example** |
| **Study Characteristics** |  |  |
| Author | The first author of the study. |  |
| Year | The year in which the study was published. |  |
| Study location | The location where the study and assessment was conducted. |  |
| Hospital | The hospital where the study and assessment was conducted. |  |
| Publication type | The venue where the study was published: peer-reviewed journal articles, book chapters, dissertations, or conference proceedings. |  |
| Sample size | The total number of study participants that took part in the study. |  |
| Male to Female ratio | The percentage of study participants that were recorded as male. |  |
| Mean age of the participants | The average age of the study participants. |  |
| Parkinson’s disease duration in years | The average number of years the study participants lived with Parkinson’s disease since the initial diagnosis. |  |
| Parkinson’s disease severity | The reported level of Parkinson’s disease severity based upon the categorised Unified Parkinson’s Disease Rating Scale |  |
| Control | The type of control used in the included study. | Healthy-age matched controls, baseline reading etc. |
| **Wearable devices characteristics** |  |  |
| Device brand | What is the name of the wearable device? | Opal, Parkinson's KinetiGraph, AX3 etc. |
| Manufacturer | What is the name of the company that produced the wearable device? | APDM, Axivity, Global Kinetics etc. |
| Wearable device status | Is the wearable device a prototype or is it an already available commercial device (e.g. Opal, Parkinson's KinetiGraph, AX3)? | Prototype, Commercial etc. |
| Regulatory approval for clinical use | Has the wearable device been clinical approved by any regulatory bodies? | FDA (Food Drug Administration), CE (Conformité Européene) medical mark etc. |
| Wearable device aim | What is the wearable device doing in the study? | Monitoring disease symptoms, monitoring biomarkers, diagnosing, making alerts, making predictions etc. |
| Sensor | What sensors are embedded in the wearable device? | Accelerometer, gyroscope etc. |
| Wearable device type | In which form is the wearable device available? | Body sensor, inertial measurement device, eye tracker etc. |
| Placement of the wearable device | Where the wearable device is worn during the experiment in paper or normally? | Wrist, chest, head, ears, forehead, eyes, fingers, foot etc. |
| Measured biosignals | What are the biosignals measured by the wearable device? | Heart rate, EEG, ECG, step counts, body temperature, blood pressure etc. |
| Connectivity | How data is transferred from the wearable device to the data storage device? | Bluetooth, WiFi etc. |
| Host device | What is the end gate device that the wearable is synchronised with/ that stores data collected by the wearable device? | Smartphone, tablet, database server, PC etc. |
| Sensing approach | How is the data being collected by the wearable device with or without the knowledge of the user? | Participatory approach (manually added and non-intrusive) where the data is collected with the user’s input.  Opportunistic approach (automatic mechanisms for collecting data). |
| Length of time the wearable device was used | How long was the wearable device used in the study? | Single session, multiple sessions over time etc. |
| Artificial Intelligence/Machine Learning technology used | What type of AI/ML algorithms are used by the researchers? | Black box, Least Absolute Shrinkage and Selection Operator, Partial least square discriminant analysis, Random forest etc. |
| Clinical aim of Artificial Intelligence/Machine Learning | What was the purpose of using Artificial Intelligence/Machine Learning in the study? | Gait analysis, Parkinson’s disease classification, ranking Parkinson’s disease symptoms etc. |
